# Strong anisotropic lifetime orientation distributions of a two-level quantum emitter around a plasmonic nanorod

**DOI:** 10.1186/1556-276X-9-194

**Published:** 2014-04-28

**Authors:** Jia-Ming Liu, Jing-Feng Liu, Yi-Cong Yu, Ling-Yu Zeng, Xue-Hua Wang

**Affiliations:** 1 State Key Laboratory of Optoelectronic Materials and Technologies, School of Physics and Engineering, Sun Yat-sen University, Guangzhou 510275, China; 2 College of Science, South China Agriculture University, Guangzhou 510642, China; 3 Department of Physics, Foshan University, Foshan 528000, China

**Keywords:** Surface plasmons, Spontaneous emission, Lifetime distribution, Nanorod, 78.67.Qa, 73.20.Mf, 42.50.-p

## Abstract

Spontaneous emission lifetime orientation distributions of a two-level quantum emitter in metallic nanorod structures are theoretically investigated by the rigorous electromagnetic Green function method. It was found that spontaneous emission lifetime strongly depended on the transition dipole orientation and the position of the emitter. The anisotropic factor defined as the ratio between the maximum and minimum values of the lifetimes along different dipole orientations can reach up to 10^3^. It is much larger than those in dielectric structures which are only several times usually. Our results show that the localized plasmonic resonance effect provides a new degree of freedom to effectively control spontaneous emission by the dipole orientation of the quantum emitters.

## Background

Spontaneous emission (SE) control of quantum emitters (QEs) is of great importance in basic quantum optics researches and new type of quantum information devices design due to its diverse range of applications such as solar energy harvesting [1,2], light-emitting diodes [3,4], miniature lasers [5,6], and single-photon source for quantum information science [7,8].

It is well known that, the spontaneous emission lifetime of QEs can be strongly modulated by the surrounding environment. So, various photonic systems, such as microcavities [9,10] and photonic crystals [11-13], have been proposed to manipulate the lifetime of QEs. Recently, metallic nanostructures have attracted extensive of interest as they support surface plasmonic resonances, which are the collective oscillations of the electron gas in metals [14,15]. Surface plasmons may greatly enhance the local electromagnetic field that leads to nanoscale ‘hot spots’ [16,17]. Such local enhancement capability enables the quantum control of the SE process at nanoscale [18-23]. An important advantage of controlling SE of QEs is its wide range of application. In [24], the SE enhancement of a single quantum dot coupled to silver nanowire was successfully measured. Such measurements proved that the SE exhibits antibunching. This means that plasmonic nanowires can provide single-photon sources, as has been demonstrated in [25] by using NV centers. Besides, alternative plasmonic systems have been presented to manipulate SE enhancement, such as hybrid waveguide [26] and plasmonic resonators [27]. Moreover, the efficient coupling between single emitter and the propagating plasmonic modes enables the realization of single photon transistor devices [28,29]. However, the investigation of SE control with different transition dipole orientations of a QE is still a challenging task. To date, no clear picture has emerged of the orientation-dependent characteristics around the metallic particles but it is of great importance in the research of interaction between light and matter [30].

In this paper, we investigate the SE lifetime of a two-level QE with different dipole moment orientations around a plasmonic nanorod. Using the Finite Element Method, we calculate the SE lifetime, anisotropic factor and find that the SE lifetime has strongly orientation dependent character which is different from the structures reported before in photonic crystals and dielectric sphere structures [11,31,32].

## Methods

In this manuscript, we only consider the case of weak QE-field coupling regime. In this regime, the SE decay lifetimes for both homogeneous and inhomogeneous environment are calculated by the formula [32-34]

(1) τr→,ω,μ→=2ω2ℏϵ0c2μ→*·ImG↔r→,r→,ω·μ→-1

where ω is the angular frequency, *c* is the speed of light in vacuum, $\overset{\to }{\mu }$ is the unit vector of the dipole moment ImG↔r→,r→,ω stands for the imaginary part of Green's tensor, and $\overset{\to }{r}$ is the position of the QE. Notice that the SE lifetime depends on the dipole orientation. As is known that the quantity ImG↔r→,r→,ω in vacuum equals $\omega \overset{↔}{I}/\left(6\pi c\right)$, where $\overset{↔}{I}$ is a unit tensor. We can easily deduce the SE lifetime *τ*_vac_(*ω*) = [*ω*^3^*d*^ 2^/(3*πℏϵ*_0_*c*^3^)]^- 1^ of QE embedded in vacuum according to Equation 1. Then, the normalized orientation-dependent SE lifetime could be defined as $\overset{˜}{\tau }\left(\overset{\to }{r},\omega ,\overset{\to }{\mu }\right)=\tau \left(\overset{\to }{r},\omega ,\overset{\to }{\mu }\right)/\tau_{\text{vac}}\left(\omega \right)$. To evaluate the difference degree of the lifetime orientation distribution, we define the anisotropic factor as

(2) ηr→,ω=τmaxr→,ω,μ→τminr→,ω,μ→'

The Green tensor in Equation 1 satisfies

(3) $$\left[\nabla \times \nabla \times -\epsilon \left(\overset{\to }{r},\omega \right)\frac{\omega^{2}}{c^{2}}]\overset{↔}{G}\left(\overset{\to }{r},{\overset{\to }{r}}^{\prime },\omega \right)=\overset{↔}{I}\delta (\overset{\to }{r},{\overset{\to }{r}}^{\prime }\right]$$

where *ϵ* is the relative permittivity. It could be calculated from the electric field of a dipole source as [35,36]

(4) $$\overset{\to }{E}\left(\overset{\to }{r},\omega \right)=\frac{\omega^{2}}{\epsilon_{0}c^{2}}\overset{↔}{G}\left(\overset{\to }{r},\overset{\to }{r},\omega \right)\cdot \overset{\to }{d}\left(\overset{\to }{r},\omega \right)$$

where $\overset{\to }{d}\left(\overset{\to }{r},\omega \right)=\overset{\to }{d}\left(\omega \right)\delta \left(\overset{\to }{r}-{\overset{\to }{r}}_{0}\right)$ is a dipole source at position ${\overset{\to }{r}}_{0}$. The whole elements of the Green tensor could be attained after setting the dipole source with *x*, *y*, and *z* polarizations in turn.

## Results and discussion

**Figure 1:**
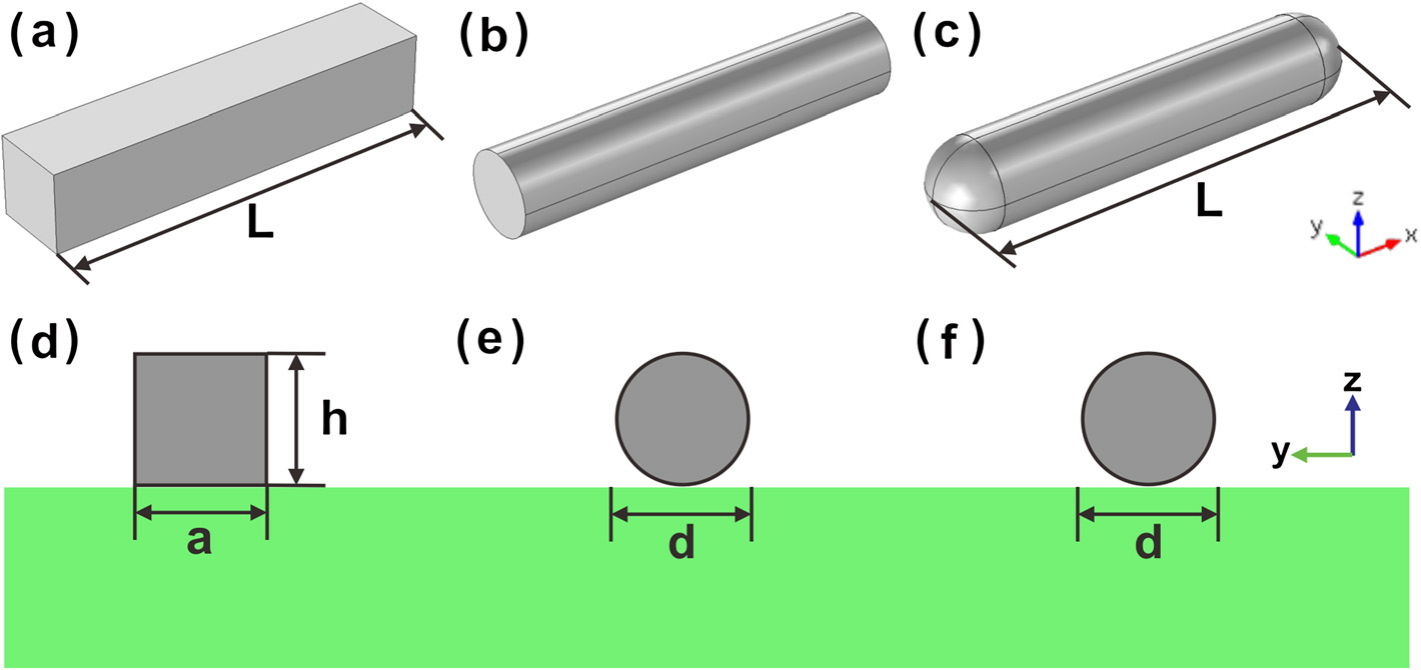
**Schematic diagrams of the gold nanorod structures. (a)** Rectangular, **(b)** cylinder, and **(c)** capsule nanorods. **(d, e, f)** The cross sections corresponding to **(a, b, c)**, respectively.

In this paper, the dielectric constant of the gold nanorod is obtained by fitting the experimental data from Johnson and Christy with piecewise cubic interpolation [37]. The nanorod is placed upon the SiO_2_ substrate with refractive index of 1.5. Other parts are set as vacuum. We consider rectangular, cylinder, and capsule nanorods in the simulations. The corresponding schematic diagrams of the structures are shown in Figure 1a,b,c, respectively. The cross sections of each structure at *x* = 0 plane are shown in Figure 1d,e,f, respectively. The width of the rectangular nanorod is *a* = 20 nm, the length is *L* = 120 nm, and the height is *h* = 20 nm. The diameter of the cylinder nanorod is *d* = 20 nm and the length is also *L* = 120 nm. The capsule nanorod is modified from the cylinder shape nanorod by changing the two ends into a half-sphere shape. The total length of the capsule-shaped nanorod is still *L* = 120 nm. We perform the simulations by the Finite Element Method with the help of the software COMSOL Multiphysics. The coordinate origin is set at the center of the nanorod, and the nanorod is placed along the *x* axis. We adopt the perfectly matched layer (PML) for the absorption boundary.

**Figure 2:**
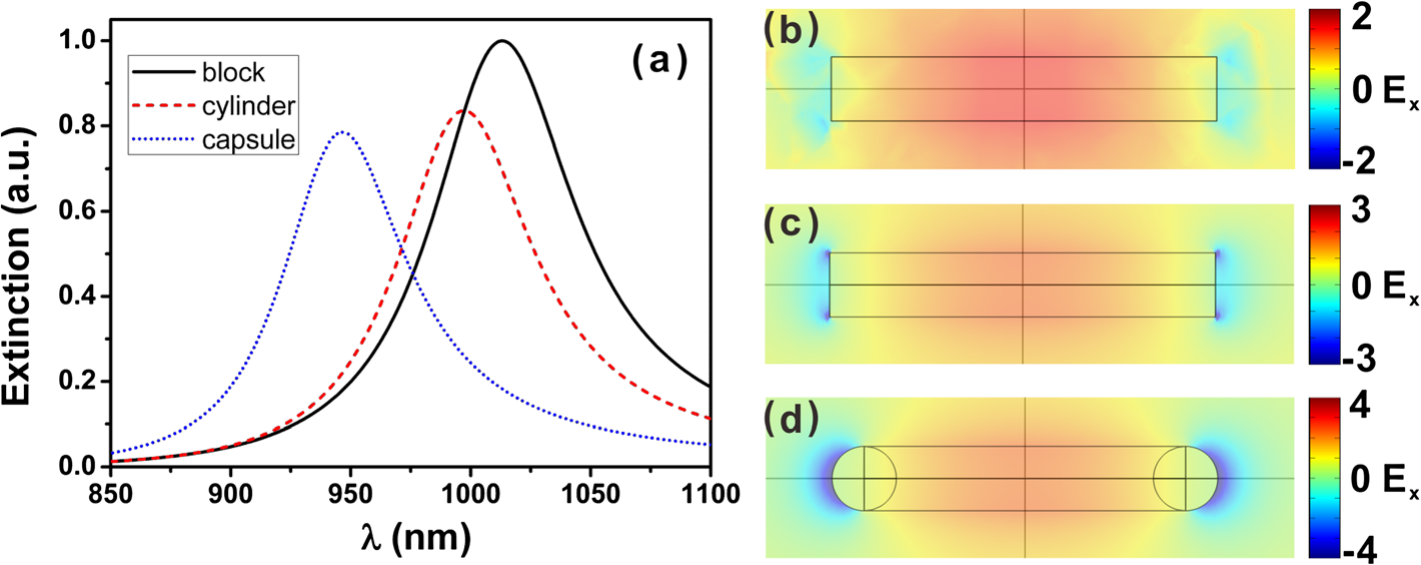
**Extinction spectra (a) of rectangular, cylinder, capsule nanorod and distributions of *x *component of electric field (b, c, d).***z =* 0 plane of the rectangular, cylinder, and capsule nanorods at wavelengths 1,013, 997, 946 nm, respectively.

In order to calculate for the plasmonic resonance frequency, we consider a planewave normal incident with *x* polarization as $\overset{\to }{E}={\overset{\to }{e}}_{x}\;\text{exp}\;\left\{-ik_{0}z\right\}$, where *k*_0_ is the wave number in vacuum. The extinction spectrums of the rectangular, cylinder, and capsule-shaped nanorods are indicated in black, red-dashed, and blue-dotted curves in Figure 2a, respectively. We observe the peaks at wavelength of 1,013, 997, and 946 nm for the rectangular, cylinder, and capsule nanorods, respectively. The plasmonic resonance wavelengths shift and the peak values vary a little for different nanorods. The corresponding distributions of the *x* component of electric field at *z* = 0 plane are shown in Figure 2b,c,d, respectively. The *x* component of electric field retains the same sign in the nanorod, which means the charges between the two ends of the nanorod are opposite, indicating an electric dipole mode [38].

**Figure 3:**
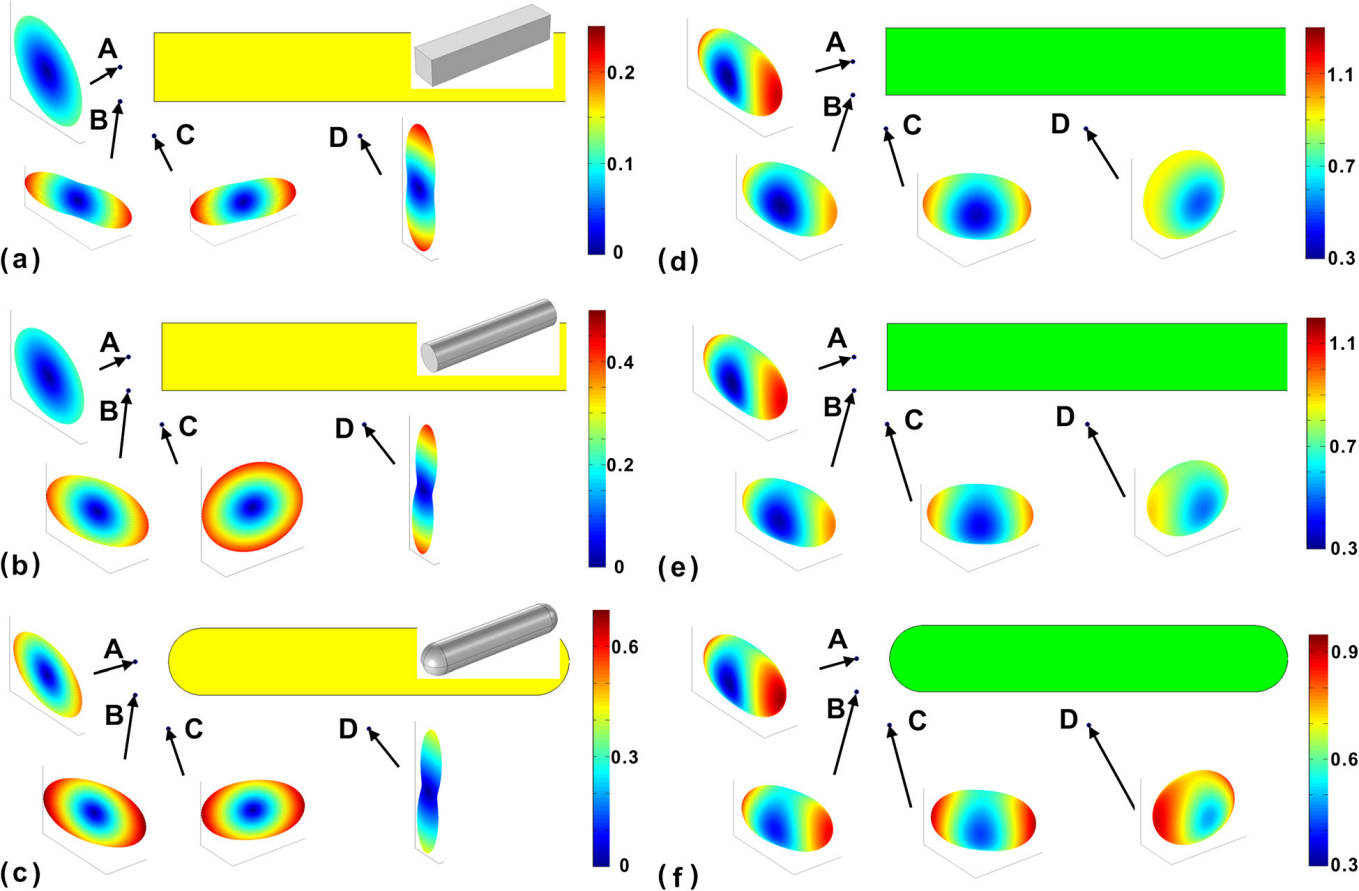
**Lifetime orientation distributions of QEs around (a, d) rectangular, (b, e) cylinder, and (c, f) capsule gold and Si nanorods.** The gold nanorods have wavelengths **(a)** 1,013, **(b)** 997, and **(c)** 946 nm, respectively. Four typical points are chosen: A (-70,0,0), B (-70,-10,0), C (-60,-20,0), and D (0,-20,0) nm. The lifetime orientation distributions of QEs around the rectangular, cylinder, capsule Si nanorods at wavelengths **(d)** 1,013, **(e)** 997, **(f)** 946 nm, respectively.

Then, we study the orientation-dependent lifetime distributions around the nanorods at the corresponding plasmonic resonance wavelengths. The orientation distributions around the rectangular, cylinder, and capsule nanorods at wavelengths of 1,013*,* 997*,* and 946 nm are shown in Figure 3a,b,c, respectively. We select four typical points A (-70,0,0) nm, B (-70,-10,0) nm, C (-60,-20,0) nm, and D (0,-20,0) nm for instance. The black arrows are the guides for the lifetime orientation distributions at these points. The yellow area is the cross section of the nanorod at *z* = 0 plane. The three-dimensional view of the nanorod is inset at the top-right position. The red color corresponds to the long lifetime, while the blue color corresponds to the short lifetime. The lifetime of the emitter has been normalized with that of the vacuum. We find that the maximum of the color bar is smaller than 1. So in all dipole directions, the lifetime of the emitters around the gold nanorods are shorter than that of the vacuum. The lifetime orientation distributions of the QE in the considered structures seem to be pancake-like with a sunken center but with different contours. It illustrates that the SE lifetime strongly depended on the direction of the transition dipole. This phenomenon is due to the localized surface plasmons which are longitudinal dipolar modes at these wavelengths. When the transition dipole moment of the QE is parallel to the electric field's direction of the longitudinal dipolar plasmon mode, the interaction between the QE and the plasmonic mode is the strongest, which leads to the shortest lifetime of the QE. The anisotropy of the lifetime distribution of the QE at point A around the capsule nanorod is larger than those around the rectangular and cylinder nanorods. This is because the end of the capsule nanorod is sharper than that of the other two nanorods, which results in the stronger field enhancement around the ends. At points B and C, the lifetime orientation distributions of the QEs are different for these nanorods. At point D, the lifetime orientation distributions of the QEs are similar for the cylinder and capsule nanorods, but different for the rectangular nanorod. This is because the sides of cylinder and capsule nanorods are round but the side of rectangular nanorod is flat.

As written in the Methods part, we define the anisotropy factor *η* to evaluate the orientation anisotropy by the ratio of the maximum lifetime over the minimum lifetime in all dipole orientations. The results of rectangular, cylinder, and capsule nanorods are shown in Table 1. The lifetime differs hundreds of times around the end of the rectangular nanorod. The orientation anisotropy of the cylinder nanorod is much stronger than that of the rectangular nanorod. The orientation anisotropy of the capsule nanorod is the strongest, and the anisotropy factor reaches up to three orders of magnitude when the emitter is placed 10 nm to the end of the capsule nanorod.

**Table 1:** **Anisotropy factor *η *** **at different positions around gold nanorod**

	**A**	**B**	**C**	**D**
Rectangular	206	386	361	60.1
Cylinder	615	858	749	126
Capsule	1,016	837	794	137

In order to underline the effect of the localized surface plasmon, we consider dielectric nanorods with the same geometrical parameters but without plasmonic modes. The material of the dielectric nanorod is chosen as Si with refractive index of 3.4. The orientation distributions around the rectangular, cylinder, and capsule dielectric nanorods at wavelengths 1,013, 997, and 946 nm are shown in Figure 3d,e,f, respectively. The green area is the cross section of the Si nanorod at *z* = 0 plane. We select the four typical points as before. We observe that the maximum of the color bar can be larger than 1. So in some dipole directions, the lifetimes of QEs will be longer than those of the vacuum. They are different from the lifetimes of the QE around the metallic nanorod. The anisotropy factors of the rectangular, cylinder and capsule-shaped dielectric nanorod are shown in Table 2. The lifetime differs only several times. The lifetime orientation anisotropy factors are much smaller than the metallic nanorod case.

**Table 2:** **Anisotropy factor *η *** **at different positions around Si nanorod**

	**A**	**B**	**C**	**D**
Rectangular	4.18	3.47	3.02	1.87
Cylinder	3.78	2.94	2.53	1.78
Capsule	2.96	2.30	2.21	1.85

**Figure 4:**
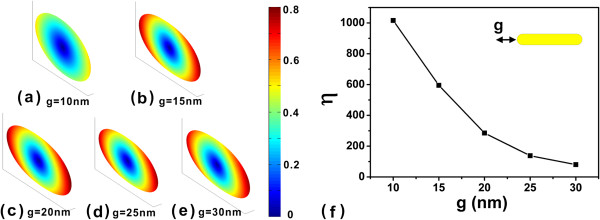
**Lifetime orientation distributions of QEs and anisotropic factor.** The distances are **(a)** 10, **(b)** 15, **(c)** 20, **(d)** 25, **(e)** 30 nm to the end of capsule-shaped nanorod at wavelength 946 nm. **(f)** The anisotropic factor at different distances.

In the following, we further study the detailed lifetime orientation distributions of the QE near the end of the capsule gold nanorod. The orientation distributions at distance *g* = 10, 15, 20, 25, and 30 nm to the end of the capsule nanorod at wavelength 946 nm is shown in Figure 4a,b,c,d,e, respectively. The orientation anisotropy factors are shown in Figure 4f. The orientation anisotropy factor reduces as the distance increases. This is because the plasmonic resonance is weakly excited when the QE is far from the nanorod.

**Figure 5:**
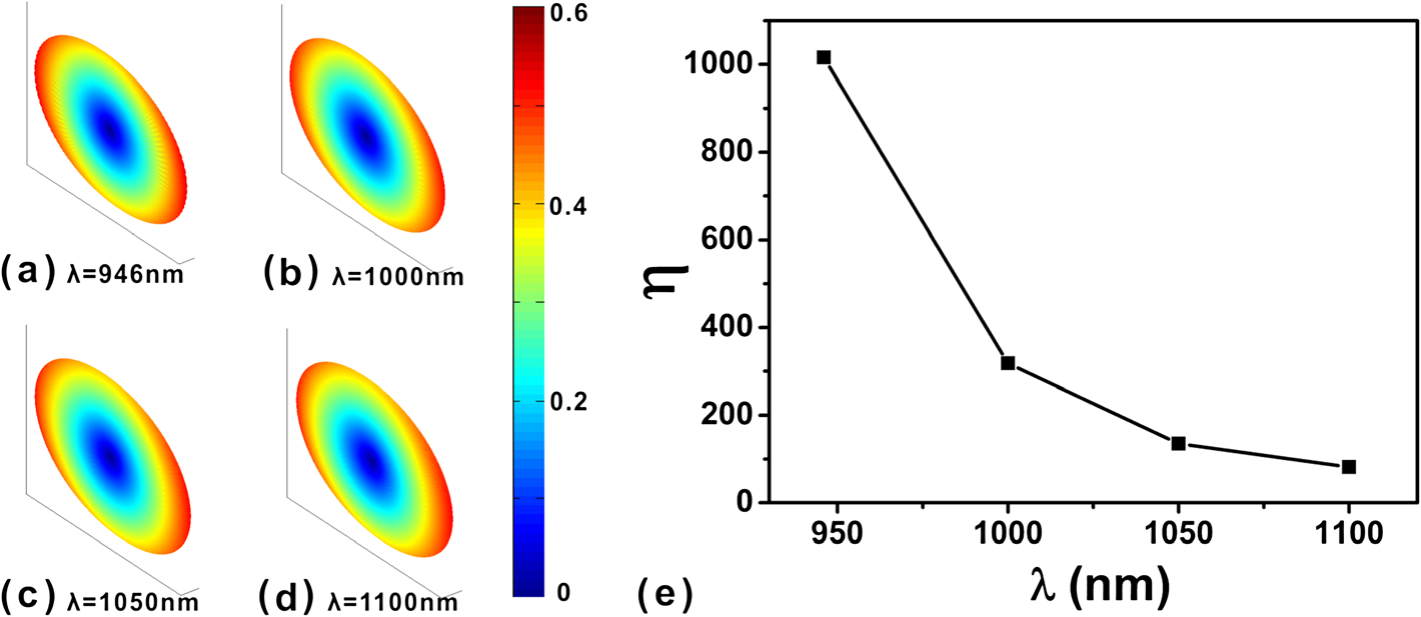
**Lifetime orientation distributions of QEs with distance 10 nm to end of capsule-shaped nanorod and anisotropic factor.** The wavelengths are **(a)** 946, **(b)** 1,000, **(c)** 1,050, and **(d)** 1,100 nm. **(e)** The anisotropic factor at different wavelengths.

Next, we consider the frequency dependence of the orientation anisotropy. We still take the capsule nanorod as example. The QE is set at (-70,0,0) nm, 10 nm apart from the end of the nanorod. The orientation distributions of the QE at wavelengths 946, 1,000, 1,050, and 1,100 nm are shown in Figure 5a,b,c,d, respectively. The orientation anisotropy factors are shown in Figure 5e. We find that the orientation anisotropy factor reduces as the wavelength moves farther away from the peak wavelength. The reduction of the orientation anisotropy factor is because the plasmon mode is weakly excited when the wavelength is moving away from the central peak frequency.

**Figure 6:**
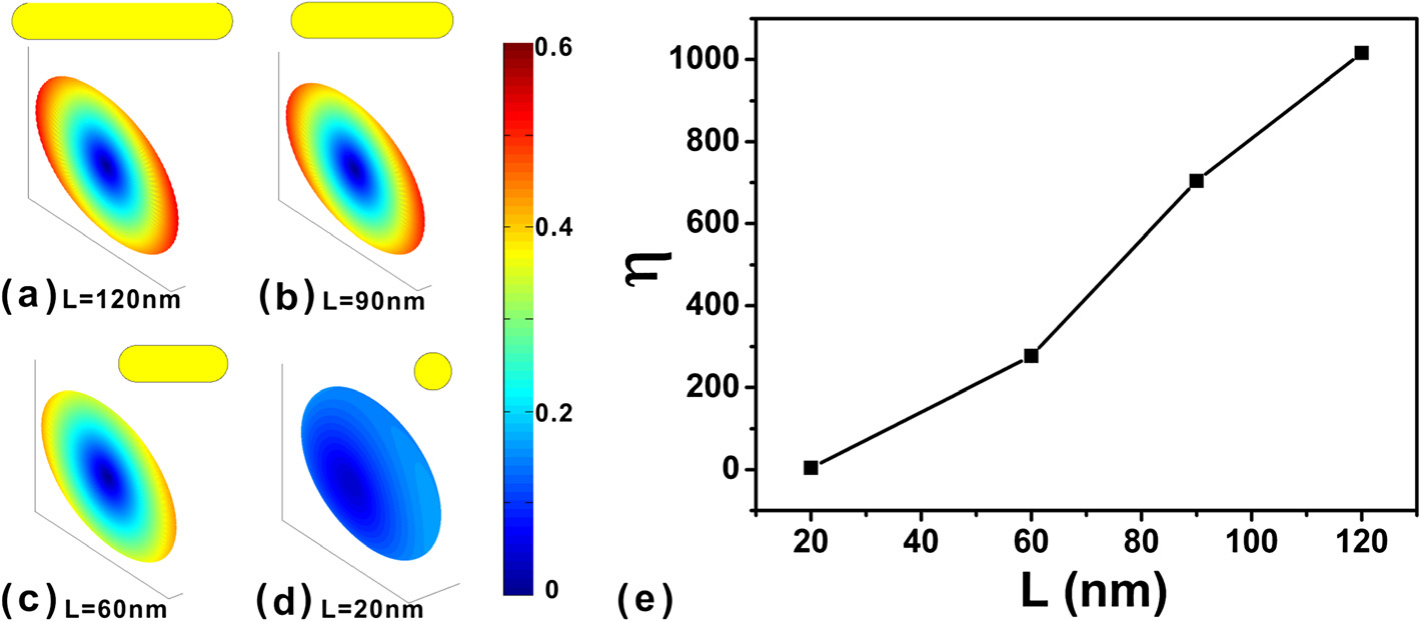
**Lifetime orientation distributions of QEs with distance 10 nm to end of capsule nanorod and anisotropic factor.** The wavelengths are 946, 791, 644, and 389 nm with nanorod lengths are *L* = **(a)** 120, **(b)** 90, **(c)** 60, and **(d)** 20 nm, respectively. The nanorod turns into sphere at the case of *L* = 20 nm. **(e)** The anisotropic factor with different length of the nanorod.

At last, we study the nanorod length dependence of orientation anisotropy. The orientation distributions of the QE at the distance 10 nm apart from the end of the capsule nanorod with length *L* = 120, 90, 60, and 20 nm are shown in Figure 6a,b,c,d, respectively. In the case of *L* = 20 nm, the nanorod turns into a sphere. The dipole plasmonic mode of nanorods with length *L* = 120, 90, 60, and 20 nm are at wavelengths 946, 791, 644, and 389 nm, respectively. The extinction spectrums of different nanorod lengths are not shown here. The orientation anisotropy factors are shown in Figure 6e. The orientation anisotropy is reduced rapidly as the nanorod length reduced.

## Conclusions

In summary, we have studied the SE lifetime orientation distributions around a metallic nanorod by using the rigorous electromagnetic Green function method. Rectangular, cylinder, and capsule nanorods are considered. The anisotropic factor near the end of the gold capsule nanorod can reach up to 10^3^. By comparing the results of a dielectric nanorod, we point out the importance of localized plasmonic resonance to the lifetime orientation anisotropy distributions. The factors of QEs position, frequency, and the length of nanorod are investigated in detail. Our results show that the localized plasmonic resonance effect provides a new degree of freedom to effectively control spontaneous emission by the dipole orientation of the QEs.

## Abbreviations

SE: spontaneous emission; QE: quantum emitter.

## Competing interests

The authors declare that they have no competing interests.

## Authors’ contributions

JML participated in the derivation of equations, performed the numerical simulations, interpreted the simulation results, and drafted the manuscript. JFL participated in the derivation of the equation and revised the manuscript. YCY participated in the analysis of the simulation results and revised the manuscript. LYZ revised the manuscript. XHW conceived of the study and revised the manuscript substantially. All authors had read and approved the final manuscript.
